# Characterization and Comparative Analysis of Olfactory Receptor Co-Receptor Orco Orthologs Among Five Mirid Bug Species

**DOI:** 10.3389/fphys.2018.00158

**Published:** 2018-03-05

**Authors:** Qi Wang, Qian Wang, Yan-Le Zhou, Shuang Shan, Huan-Huan Cui, Yong Xiao, Kun Dong, Adel Khashaveh, Liang Sun, Yong-Jun Zhang

**Affiliations:** ^1^State Key Laboratory for Biology of Plant Diseases and Insect Pests, Institute of Plant Protection, Chinese Academy of Agricultural Sciences, Beijing, China; ^2^Department of Plant Protection, College of Horticulture and Plant Protection, Yangzhou University, Yangzhou, China; ^3^DanDong Entry-Exit Inspection and Quarantine Bureau, Dandong, China; ^4^Key Laboratory of Tea Quality and Safety Control, Ministry of Agriculture, Tea Research Institute, Chinese Academy of Agricultural Sciences, Hangzhou, China

**Keywords:** olfactory receptor co-receptor, mirid bugs, gene structure, sequence analysis, evolution analysis

## Abstract

The phytophagous mirid bugs of *Apolygus lucorum, Lygus pratensis* as well as three *Adelphocoris* spp., including *Adelphocoris lineolatus, A. suturalis*, and *A. fasciaticollis* are major pests of multiple agricultural crops in China, which have distinct geographical distribution and occurrence ranges. Like many insect species, these bugs heavily rely on olfactory cues to search preferred host plants, thereby investigation on functional co-evolution and divergence of olfactory genes seems to be necessary and is of great interest. In the odorant detection pathway, olfactory receptor co-receptor (Orco) plays critical role in the perception of odors. In this study, we identified the full-length cDNA sequences encoding three putative Orcos (*AsutOrco, AfasOrco*, and *LpraOrco*) in bug species of *A. suturalis, A. fasciaticollis*, and *L. pratensis* based on homology cloning method. Next, sequence alignment, membrane topology and gene structure analysis showed that these three Orco orthologs together with previously reported AlinOrco and AlucOrco shared high amino acid identities and similar topology structure, but had different gene structure especially at the length and insertion sites of introns. Furthermore, the evolutional estimation on the ratios of non-synonymous to synonymous (Ka/Ks) revealed that Orco genes were under strong purifying selection, but the degrees of variation were significant different between genera. The results of quantitative real-time PCR experiments showed that these five Orco genes had a similar antennae-biased tissue expression pattern. Taking these data together, it is thought that Orco genes in the mirid species could share conserved olfaction roles but had different evolution rates. These findings would lay a foundation to further investigate the molecular mechanisms of evolutionary interactions between mirid bugs and their host plants, which might in turn contribute to the development of pest management strategy for mirid bugs.

## Introduction

Due to long-term adoption of transgenic Bt (*Bacillus thuringiensis*) cotton and the associated reduction in broad-spectrum insecticide used for controlling *Helicoverpa* spp. (Wu et al., 2008), several species of the mirid bugs (Hemiptera: Miridae) including *Apolygus lucorum, Lygus pratensis* as well as three *Adelphocoris* spp., including *Adelphocoris lineolatus, A. suturalis* and *A. fasciaticollis* have become most important pest species in cotton fields of northern China (Lu et al., 2010). Besides cotton, these polyphagous mirid species cause severe destructions to many other important crops including vegetables, fruits trees and tea plants (Lu and Wu, 2008). It was reported that these five mirid species are significantly different in geographic distribution and seasonal abundance in China (Lu et al., 2008a). The *A. lucorum* is widely distributed in whole China, while three *Adelphocoris* species and *L. pratensis* occur mainly in Yangtze River region and the northern parts of Yellow River region, and in the colder region of northwest China, respectively (Lu and Wu, 2008). The screening of overwintering and early season host plant ranges suggested that mirid bugs from different regions employed distinct host plant ranges to survive winter and early spring, and these differences are significantly linked to their reliance on local plants (Lu et al., 2011). Consequently, the interactions between mirid species and local host plants should play crucial roles in determining ecological landscape-level especially their different geographic distribution and seasonal abundance. A better understanding of the underlying species-preferential host plants tracking would help to define co-evolution between different mirid species and their host plants, and ultimately facilitate the development of regional forecasting and pest management strategies.

Insect olfaction plays important roles in locating host plant. Several classes of molecules including odorant binding proteins (OBPs), chemosensory proteins (CSPs), odorant receptors (ORs), sensory neuron membrane proteins (SNMPs) and odorant degradation enzymes (ODEs) play important roles in odorant signal transduction pathway (Leal, 2013). ORs located in the dendrite membrane of olfactory sensory neurons (OSNs) and are considered to play a central role in identifying the distinct odorants and activating the OSNs (Clyne et al., 1999; Hallem et al., 2004). Compared with mammal ORs, insect ORs have seven transmembrane domains (TMDs) but employ a “reversed” topology with their N-terminus inside the cell and the C-terminus exposed to the external environment (Benton et al., 2006; Lundin et al., 2007; Hull et al., 2012). To detect the odorants, ORs could interact with a conserved olfactory receptor co-receptor (Orco) and then form ligand-gated ion channels (Sato et al., 2008; Wicher et al., 2008).

Orco is previously referred to as OR83b in *Drosophila melanogaster*, OR2 in *Bombyx mori*, and OR7 in mosquito species (Vosshall and Hansson, 2011). Conventional ORs demonstrate low sequence identity, whereas Orco is strikingly well conserved across insect species. It was reported that Orco has no direct relation with odor binding or discrimination (Nichols and Luetje, 2010; Nichols et al., 2011), but is essential for ion channel formation and olfactory cues transduction. In fact, Orco could interact with conventional ORs to form heterodimeric complexes, whereas conventional ORs were responsible for specifically binding to structurally diverse odorants (Larsson et al., 2004; Benton et al., 2006). Also, Orco was confirmed to be activated by VUAA1 as a functional ion channel in homomeric complex, even in the absence of conventional olfactory receptors (Jones et al., 2011). However, VU0183254, one of the analogs of VUAA1, showed the ability to “lock” hemomeric and homomeric ion channels in a non-competitive way due to its affinity to Orco (Jones et al., 2012). Coincidentally, these functional hemomeric and homomeric channels can be also blocked by amiloride derivatives when they were activated (Pask et al., 2013). Disruption in the transcript expression of Orco could significantly impair olfactory behavior responses in all the tested insect species, including *D. melanogaster* (Larsson et al., 2004), *Acyrthosiphon pisum* (Zhang et al., 2017), *Locust amigratoria* (Li et al., 2016), *Spodoptera litura* (Dong et al., 2013), *Lymantria dispar* (Lin et al., 2015), *Aedes aegypti* (DeGennaro et al., 2013), *Microplitis mediator* (Li et al., 2012), *A. lucorum* (Zhou et al., 2014), and *Bactrocera dorsalis* (Zheng et al., 2012). Due to the crucial role in olfactory perception, Orco is known as an excellent target for investigating co-evolution across sibling insect species (Lu et al., 2009).

The plant mirid species of *Lygus* spp., *Adelphocoris* spp., and other species strongly rely on olfactory cues to regulate their chemical perception behaviors. Series of studies on chemoreception of plant mirids were reported such as antennal morphological and electrophysiological characteristic (Chinta et al., 1997; Sun et al., 2014a), putative odorants (Koczor et al., 2012; Sun et al., 2013), physiological functions of OBPs (Gu et al., 2011; Hull et al., 2014; Sun et al., 2014b) and conventional ORs (Yan et al., 2015; An et al., 2016; Xiao et al., 2016; Zhang et al., 2016). In the current study, we focused on the evolutionary divergence of Orco orthologs among plant bug species from distinct geographic regions of China. Three Orco genes from *A. suturalis, A. fasciaticollis* and *L. pratensis* are were newly identified. Gene structures, substitution rates and tissues-biased expression of Orco orthologs from five bug species were investigated to further figure out the evolutionary divergence in different mirid bugs.

## Materials and methods

### Insect collection and rearing

Five mirid bug species including *A. lucorum, L. pratensis, A. lineolatus, A. suturalis* and *A. fasciaticollis* were collected from cotton fields at Langfang (Latitude 39.53°N, Longitude 116.70°E) or Kuerle (Latitude 41.45°N, Longitude 85.48°E) experimental station of the Chinese Academy of Agricultural Sciences. The laboratory colony was kept in 20 × 10 × 6 cm rearing containers and was reared on green beans (*Phaseolus vulgaris* L.) and a 10% sucrose solution (Lu et al., 2008b). Green beans also served as the oviposition substrate and were changed every other day. Beans containing eggs were subsequently placed in rearing containers lined with filter paper. After the emergence of the nymphs, the individuals were transferred to identical containers that were covered with nylon organdy mesh to allow air circulation. The nymphs were provided with fresh food every 2 d until the emergence of adults. Each container housed approximately 100 nymphs or 60 adults. The laboratory colony was maintained at 29 ± 1°C, 60 ± 5% relative humidity (RH), and 14 h:10 h light: dark (L: D) photoperiod.

### RNA extraction and cDNA synthesis

Antennae from newly eclosion adults were excised and immediately frozen in liquid nitrogen, then stored at −80°C until use. Total RNA was isolated by Trizol reagent (Invitrogen, Carlsbad, CA, USA) following the manufacturer's instructions. The RNA quantity and integrity were checked using 1.2% agarose gel electrophoresis and a NanoDrop 2000 spectrophotometer (NanoDrop, Wilmington, DE, USA). Total RNA was treated with RQ1 RNase-Free DNase (Promega, Madison, USA) at 37°C for 30 min to remove residual DNA. The cDNAs were synthesized using the Superscript III Reverse Transcriptase system (Invitrogen, Carlsbad, CA).

### Gene cloning and sequence analysis

*AsutOrco, AfasOrco*, and *LpraOrco* genes were cloned using degenerate primers (Table S1). Each reaction contained 300 ng antennal cDNA and 0.5 units of Ex Taq DNA Polymerase (TaKaRa, Dalian, China). The cycling parameters were: 95°C for 2 min followed by 35 cycles at 94°C for 30 s, 55°C for 30 s, 72°C for 60 s, and final extension at 72°C for 10 min. The PCR product was gel-purified and sub-cloned into the pEASY-T3 vector (TransGen, Beijing, China) and then sequencing validation was performed. The 5′ and 3′ regions of Orco genes were amplified using SMARTer™ RACE cDNA amplification kit (Clontech, Mountain View, CA, USA) using gene-specific primers (GSP) (Table S1). Touchdown PCR was performed as follows: 95°C for 2 min followed by 5 cycles at 94°C for 30 s, 72°C for 2 min; 5 cycles at 94°C for 30 s, 70°C for 30 s, and 72°C for 90 s, 30 cycles at 94°C for 30 s, 68°C for 30 s, and 72°C for 90 s; and a final 10 min incubation at 72°C. The RACE PCR products were sub-cloned into the pEASY-T3 vector (Transgene, Beijing, China) and then sequenced. The full-length Orco genes were confirmed with LA Taq DNA polymerase (Takara, Dalian, China) by PCR using gene-specific primers (Table S1).

The full length Orco sequences were aligned by ClustalX 2.1 and edited by GeneDoc 2.7.0 software. TOPCONS (http://topcons.cbr.su.se/) (Tsirigos et al., 2015) was used to identify the number and location of predicted transmembrane domains. The topology diagrams were constructed using TOPO2 Transmembrane Protein Display by the server at http://www.sacs.ucsf.edu/TOPO2/ (SJ)^1^.

### Gene structure and selective pressure analysis

Genomic DNAs from antennae were extracted using TIANamp genomic DNA kit (TIANGEN, Beijing, China) followed the manufacturer's instruction. Introns of Orco genes were amplified using specific primers (Tables S2–S4).The neighbor joining tree of Orco gene from various insect species were constructed using MEGA7.0 program with a p-distance model and a pairwise deletion of gaps. Bootstrapping was performed by the re-sampling amino acid positions of 1000 replicates, the synonymous and non-synonymous divergence was analyzed using modified Nei-Gojobori (Jukes-Cantor) (assumed transition/transversion bias = 1.21) method in MEGA 7.0 (Jukes and Cantor, 1969; Zhang et al., 1998; Kumar et al., 2016).

### Quantitative real-time PCR (qPCR) measurement

The expressions profiles of Orco gene in different tissues of both genders were evaluated by using qPCR measurement on an ABI Prism 7,500 Fast Detection System (Applied Biosystems, Carlsbad, CA, USA).The reference genes β-actin (GenBank accession number: GQ477013, KU230353, KF921006, KU188517, and MG397129, separately) were used as the endogenous control to normalize the target gene expression and correct for any sample-to-sample variation. The primers (Table S5) of the target and reference genes were designed by BEACON DESIGNER 7 (PREMIER Biosoft International). The specificity of each primer set was validated by melt-curve analysis, and the efficiency was calculated by analyzing standard curves with a five-fold cDNA dilution series. Each qPCR reaction was conducted in 20 μL mixture containing 10 μL of 2 × Super-Real PreMix Plus (TIANGEN, Beijing, China), 0.6 μL of each primer (10 μM), 0.4 μL of 50 × Rox Reference Dye, 1 μL of sample cDNA and 7.4 μL of sterilized H_2_O. The qPCR cycling parameters consisted of 95°C for 15 min, followed by 40 cycles of 95°C for 10 s and 62°C for 30 s, and melt curve stages at 95°C for 15 s, 60°C for 1 min, and 95°C for 15 s. The experiments for the test samples, endogenous control and negative control were performed in triplicate to ensure reproducibility. The comparative 2^−ΔΔ*CT*^ method was used to calculate the relative transcript levels in each tissue samples (Livak and Schmittgen, 2001). All of the data were normalized to endogenous β-actin levels from the same tissue samples.

## Results and discussion

### Cloning and sequence analysis of Orcos

Among the five plant bug species, two Orcos, AlinOrco from *A. lineolatus* and AlucOrco from *A. lucorum* were identified in our previous work (Zhou et al., 2014; Xiao et al., 2016). Here, we focused on other three Orco genes from *A. suturalis, A. fasciaticollis*, and *L. pratensis*. The rest three Orco genes were obtained by homology-based cloning (Hull et al. 2012) using degenerate primers (Table S1). A 400 bp fragment encoding putative Orco was amplified from *A. fasciaticollis, A. suturalis*, and *L. pratensis*, respectively. The remaining 5′and 3′ end sequences were further obtained using RACE PCR using gene specific primers. Finally, three full length sequences encoded *AfasOrco, AsutOrco*, and *LpraOrco* were assembled and deposited in GenBank with the accession numbers MF153393, MF153394, and MF153395, separately. The open reading frames (ORFs) of *AsutOrco, AfasOrco*, and *LpraOrco* were 1416, 1416, and 1422 bp, respectively, which resembled the full length of previously reported Orco genes (Hull et al., 2012; An et al., 2016; Xiao et al., 2016).

**Figure 1:**
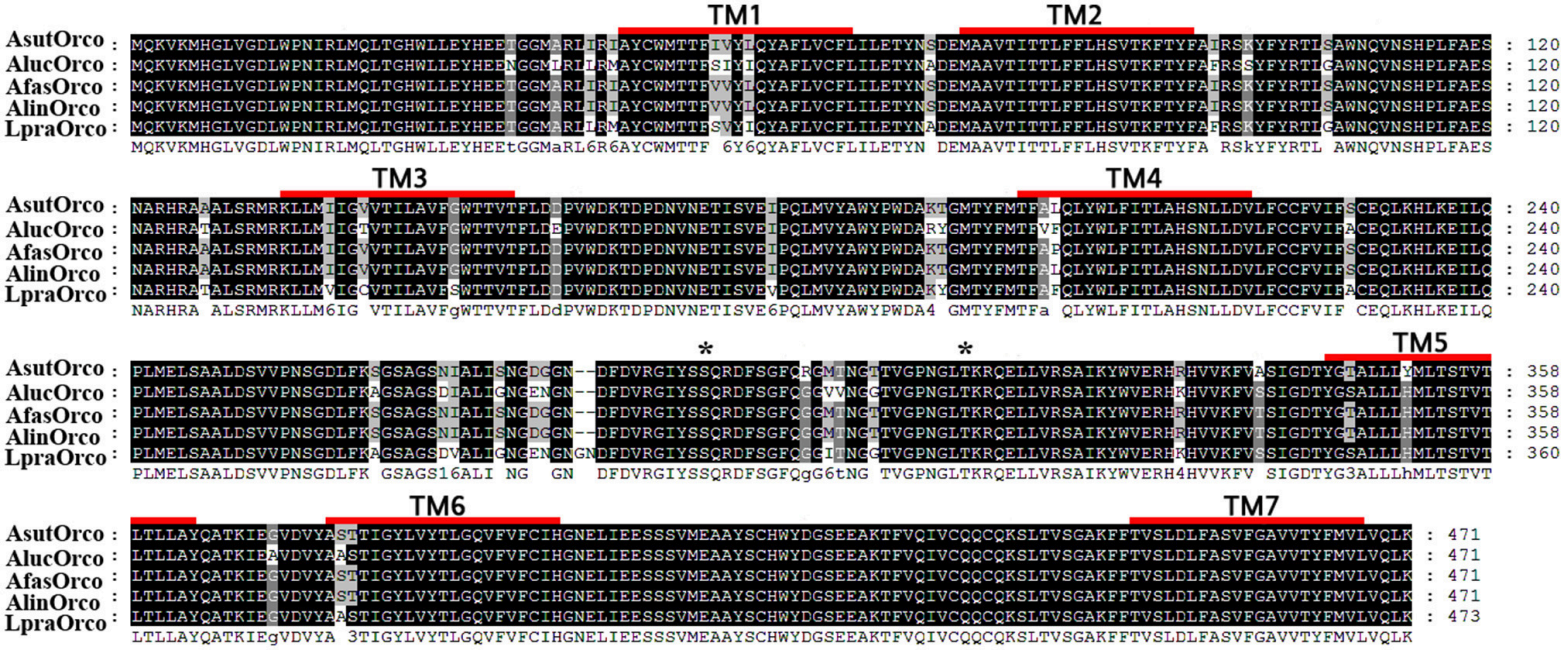
Sequence alignment of Orcos from five mirid bug species. Amino acid sequences are aligned by ClustalX 2.1 and edited by GeneDoc 2.7.0 software; the predicted positions of seven putative transmembrane domains (TM1-7) are marked with red transverse line.

Results of sequence alignment indicated that all five Orcos including AfasOrco, AsutOrco, LpraOrco, AlinOrco and AlucOrco were rather conserved across the species (Figure 1). The amino acid identity among species of genus *Adelphocoris* and even across the genera of *Adelphocoris, Lygus*, and *Apolygus* was up to 99.6 and 96.8 %, respectively (Table S6). Unlike highly conventional ORs (Clyne et al., 1999; Gao and Chess, 1999), alignment of 200 Orco amino acid sequences (Table S7) from 8 orders showed a 62.6% identity (data not shown). These findings coincide with the previous point of view that Orco is highly conserved (Krieger et al., 2003; Melo et al., 2004; Briguad et al., 2009; Zhao et al., 2013).

Generally, different regions in the gene may play different roles. A predicted algorithm based on TOPCONS revealed these five Orco shared a similar atypical seven trans-membrane topology with their N-terminus inside the cell and the C-terminus exposed to the external environment (Figure 1 and Figure S1). Consequently, the full Orco sequences can be divided into 15 regions, including the intracellular N terminal region, the seven transmembrane regions, the three intracellular loops, the three extracellular loops, and the C terminal region. These data were also consistent with the previous reports (Carraher et al., 2012; Missbach et al., 2014). The amino acid variation among different regions was significantly different with the highest variable level observed at transmembrane regions TM3 and intracellular loop 2 (IL2) that could be involved in ligands binding (Chao et al., 1999; Capendeguy et al., 2006). While no variation was found at intracellular loop 3 (IL3), TM7 and C terminus (Figures S1, S2). It was reported that IL3 participates in the channel activation interaction between conventional ORs and Orco in D. melanogaster and (Benton et al., 2006; Turner et al., 2014). As a key residue, the conserved aspartic acid in TM7 could influence the responses of Orco hemomeric and homomeric ion channels to agonist VUAA1 and odors (Kumar et al., 2013).

### Gene structures of Orcos from five bug species

**Figure 2:**
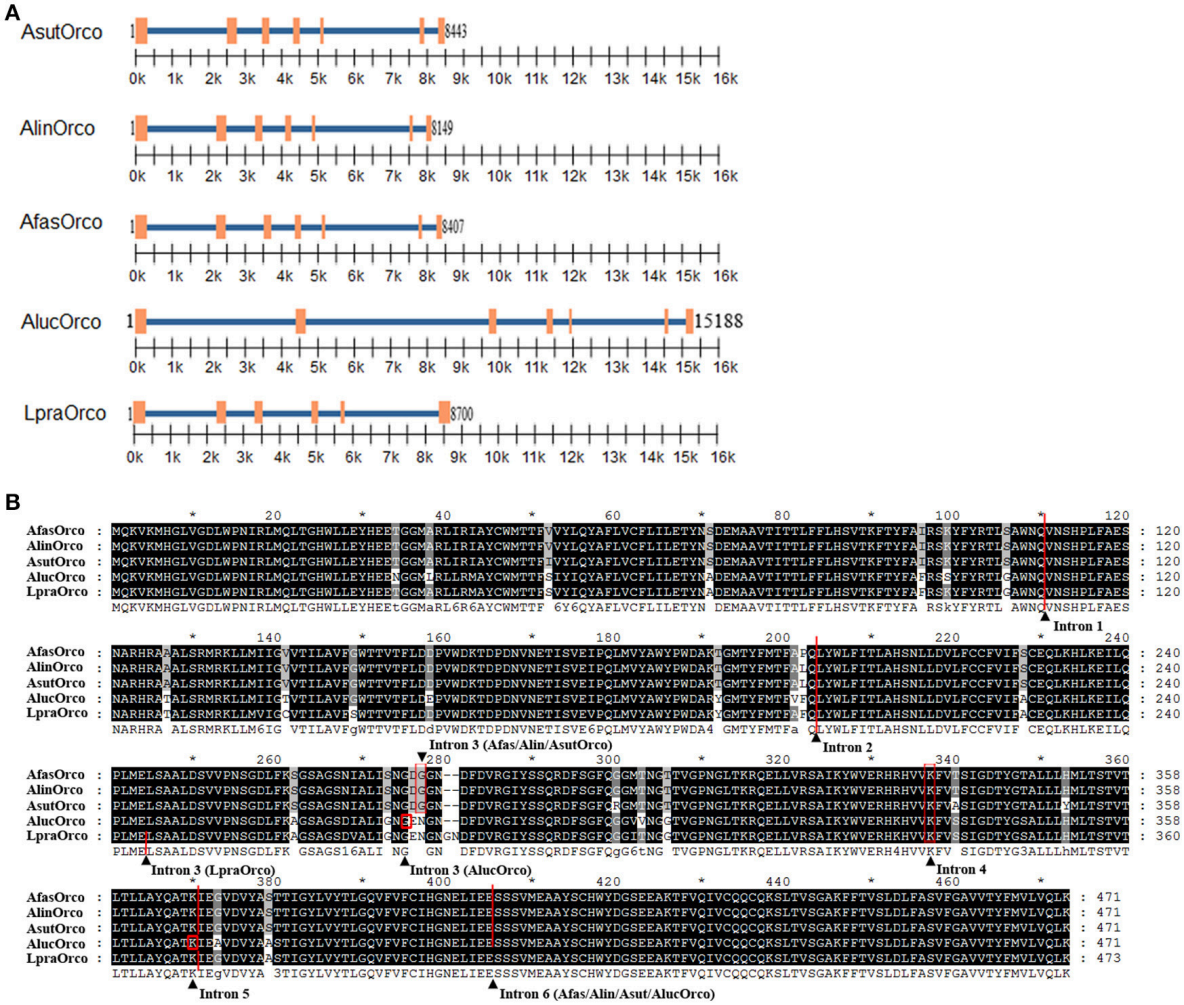
Gene structure and intron insertion loci of five Orcos. **(A)** Location of extrons (orange rectangles) and introns (blue line) in different Orco genes. **(B)** Insertion loci labeled using black triangle of different introns in Orco sequence.

Introns in Orco genes from different bug species were distinct and sequence identity of at the same position across five bug species was extremely low (about 41 %) in comparison to rather conserved amino acids (data not shown) (Figure 2). Orco within genus *Adelphocoris* shared similar seven exons, six introns and their insertion loci suggesting the most closely relationships among the three bug species. AlucOrco also had seven exons and six introns, but the length of each intron was significantly larger than that of corresponding introns from genus *Adelphocoris*. Moreover, the insertion sites of third and fourth introns were also different from Orco in genus *Adelphocoris* (Figures 2A,B). Notably, only six exons and five introns were found in LpraOrco gene, the last intron of which was lost and the third intron was located between Glu244 and Leu245.

Generally, the more intron number and larger intron length indicate a higher phylogenetic level (Nixon et al., 2002; Koonin, 2006; Wu et al., 2013; Park et al., 2014). Adult *A. lucorum* displays the most extensive distribution in China, whereas *L. pratensis* mainly occurred in Xinjiang Uygur Autonomous Region (Jiang et al., 2015). The host range is consistence with phylogenetic level among the five mirid bug species; *A. lucorum* has the widest host range including 54 families, however, *L. pratensis* merely owns 21 families (Jiang et al., 2015). Additionally, adult *A. lucorum* prefers to track better host plant food during different seasons than that of other four bug species (Pan et al., 2015; Wang et al., 2017). Likewise, olfaction especially the OR family is believed to play essential roles in the host selection for mirid bugs plant (Yan et al., 2015; Zhang et al., 2016). Therefore, our analyses indicate there might be a potential association between Orco evolution rate and the ecological adaption among these five mirid species, which could contribute to clarify the molecular mechanisms of evolutionary interactions between mirid bugs and their host plants. However, this speculation still needs to be proved by more evidences.

### Evolution analysis of Orco orthologs

**Figure 3:**
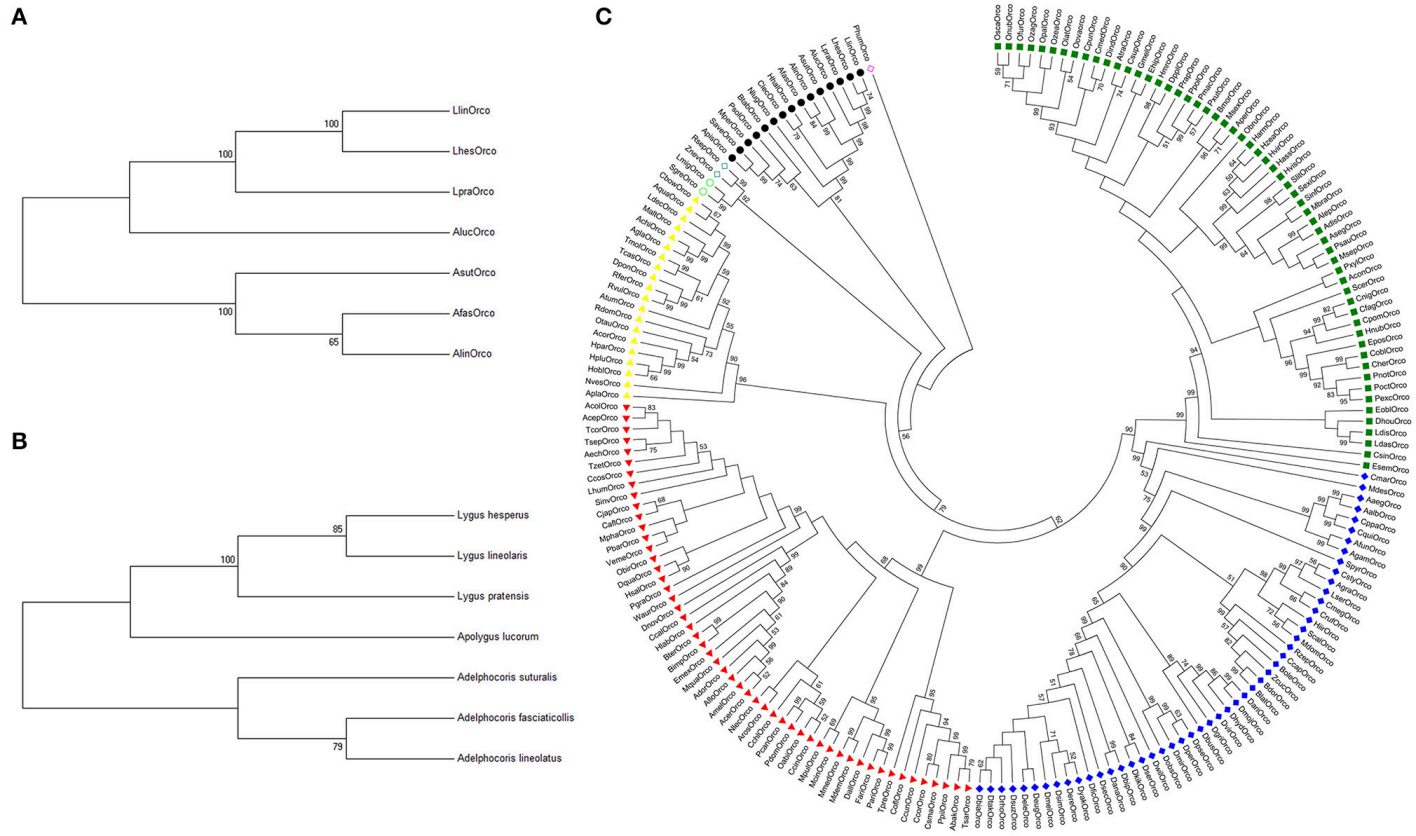
Neighbor joining tree of Orcos from different insect species. **(A)** Phylogenetic tree of Orcos from seven bug species. **(B)** Phylogenetic relationships among seven species constructed using species-specific cytochrome oxidase subunit I (*COI*). **(C)** Phylogenetic tree of insect Orcos from different orders. Yellow triangle, Coleoptera; Red triangle, Hymenoptera; Dark blue solid diamond, Diptera; Dark green solid square, Lepidoptera; Black solid circle, Hemiptera; Light blue hollow circle, Orthoptera; Light blue hollow diamond, Blattaria; Light red hollow circle, Anoplura.

There was a clear conserved orthologous relationship among AfasOrco, AsutOrco, LpraOrco, and other four bug Orcos (AlucOrco, AlinOrco, LlinOrco, LhesOrco). Phylogenetic relationship was largely consistent with the species tree constructed from the alignment of species-specific cytochrome oxidase subunit I (*COI*) (Figures 3A,B). So, we suggested that Orco was significantly conserved and could function as a molecular marker of evolution across bug species. Also relatedness analysis of these seven Orcos to the other 193 Orco sequences from eight insect orders indicated that Orco was highly conserved within insect order. Orco sequences of the same order were strictly clustered together with strong bootstrapping support (Figure 3C), indicating this phylogenetic clade was highly conserved and may fulfill conserved function.

The ratios of non-synonymous to synonymous substitutions estimated for 14 Orco genes from 5 orders were listed in Table 1. All the ratios were far less than 1.0 indicating that Orco genes are under strong purifying selection pressure. The strong purifying selection pressure suggested a functional conservation, which had been proven by substantial documents. The lack of Orco leading to a similar reduction of olfaction indicates the consistent roles in odor perceptions, suggesting the interspecific conservation of Orco indirectly (Zhou et al., 2014; Liu et al., 2017; Trible et al., 2017). Furthermore, the interspecific functional conservation has been confirmed directly by transgenic rescue experiment. The defects of olfaction in DmelOrco mutant could be rescued by transgenic expression of DmelOrco, CcapOrco, AgamOrco and HzeaOrco, respectively (Jones et al., 2005). It was also demonstrated that Orco, as an obligatory part of ligand-gated ion channel, played conservative functions in ligand binding, and was activated by the agonist VUAA1 dutifully (Benton et al., 2006; Sato et al., 2008; Jones et al., 2011).

**Table 1:** The ratio of non-synonymous to synonymous substitutions of Orco genes in five orders.

	**AlucOrco**	**AlinOrco**	**AsutOrco**	**AfasOrco**	**LpraOrco**	**LlinOrco**	**LhesOrco**	**AaegOrco**	**AgamOrco**	**DmelOrco**	**HarmOrco**	**BmorOrco**	**AmelOrco**	**MmedOrco**
AlucOrco														
AlinOrco	0.2636													
AsutOrco	0.2790	0.5936												
AfasOrco	0.2699	0.4948	0.7443											
LpraOrco	0.2198	0.2263	0.2458	0.2373										
LlinOrco	0.2351	0.2194	0.2388	0.2302	0.1502									
LhesOrco	0.2351	0.2194	0.2388	0.2302	0.1502	0								
AaegOrco	**0.4965**	**0.3992**	**0.3989**	**0.4077**	**0.4886**	**0.4966**	**0.4966**							
AgamOrco	**0.4332**	**0.3845**	**0.3964**	**0.3927**	**0.4166**	**0.4290**	**0.4290**	0.3013						
DmelOrco	**0.4562**	**0.3710**	**0.3694**	**0.3763**	**0.4524**	**0.4664**	**0.4664**	0.3229	0.3457					
HarmOrco	**0.4562**	**0.4035**	**0.4140**	**0.4151**	**0.4431**	**0.4503**	**0.4503**	0.4372	0.4216	0.3562				
BmorOrco	**0.4546**	**0.4358**	**0.4539**	**0.4446**	**0.4451**	**0.4391**	**0.4391**	0.3859	0.3869	0.2947	0.2981			
AmelOrco	**0.3657**	**0.3678**	**0.3717**	**0.3728**	**0.3680**	**0.3610**	**0.3610**	0.4414	0.4686	0.4117	0.4979	0.4154		
MmedOrco	**0.3784**	**0.2887**	**0.2999**	**0.2942**	**0.3689**	**0.3715**	**0.3715**	0.3678	0.3464	0.3343	0.3248	0.3524	0.2948	

*Estimations of synonymous substitutions and non-synonymous divergences were computed according to Modified Nei-Gojobori method (Jukes-Cantor). The ratios of non-synonymous to non-synonymous substitutions are listed in the table. Estimates in bold represented the ratio between species of other orders and mirid bugs*.

Orcos are under strongly purifying selection pressure and exhibit potential conserved olfaction roles. However, our estimation on the ratios of non-synonymous to synonymous substitutions (Ka/Ks) revealed that their levels of the purifying selection pressure significantly varied in the genera and species. Generally, the values of Ka/Ks were similar among species within same genus, but were different from species across genera. As shown in Table 1, when used DmelOrco, AgamOrco or Orco genes from other model species as outgroup, the range of Ka/Ks values of Orco genes from three *Adelphocoris* species were evaluated as (0.288–0.454), which was significantly different to that of AlucOrco (0.365–0.496) from *Apolygus* genus, or LpraOrco, LlinOrco and LhesOrco (0.361–0.497) from *Lygus* genus. These findings indicted there might be a strong constraint on functional variation within Orco from same genus, as illustrated above. In addition, these results were faultlessly correlated to the phylogenetic analyses (Figure 3). Three Orco genes from *Adelphocoris* species fall into the same clade, AlucOrco and three Orco from *Lygus* species clustered in another clade. Because of the evolutionary synchronization between Orco genes and their mirid species (Figures 3A,B), we proposed that the degrees of variation (suggested by Ka/Ks values) on Orco protein coding regions could reflected the phylogenetic levels of mirid bug species, and our data would lay a foundation on the further studies on the molecular mechanisms of speciation of mirid bugs.

### Expression profiles of five Orcos

**Figure 4:**
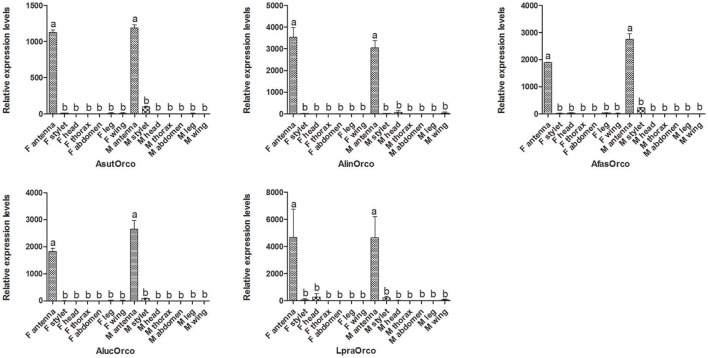
Orco expressions in different tissues of five mirid bug species. The error bars represent standard error, and different letters above each bar denote significant differences (*P* < 0.05).

In general, target gene with different tissue expressions would play different physiological function. To figure out the potential roles of Orco in mirid bugs species, qPCR measurement was conducted to assess their tissue-specific expressions (Figure 4). The results demonstrated that these five Orco genes share similar antennae-biased expression profiles, which were similar to that in *L. hesperus* (Hull et al., 2012). So, we suspected that Orco in different mirid bugs could be associated with clear olfactory roles. It was reported that silencing in *A. lucorum* of the olfactory co-receptor Orco gene by RNA interference could induce EAG response declining to two putative semiochemicals (Zhou et al., 2014). However, some Orco could be also expressed in non-olfactory organs such as proboscis and legs, suggesting that Orco might be involved in the contact chemosensory perception and could help to search hosts in close distance and perceive the status of hosts (Lu et al., 2009; Hull et al., 2012). In this study, faint transcript levels of these five Orcos were detected in stylets, legs, head and other non-olfactory organs (Figure 4) suggesting the potential roles of Orco in taste recognition of bugs. Besides in mirid bug species, Orco of *B. dorsalis* could fulfill a role involved in the perception of Rhodojaponin-III, a non-volatile compound (Yi et al., 2014).

## Author contributions

Y-JZ and LS conceived and designed the experimental plan. QiW, Y-LZ, and H-HC performed the experiments. QiW, QianW, SS, YX, KD, and AK analyzed the data. QiW and QianW drafted the manuscript. LS and Y-JZ refined and approved the final manuscript.

### Conflict of interest statement

The authors declare that the research was conducted in the absence of any commercial or financial relationships that could be construed as a potential conflict of interest.

## Footnotes

^1^S.J., J., TOPO2, Transmembrane protein display software. http://www.sacs.ucsf.edu/cgi-bin/open-topo2.py.

## Supplementary material

Figure S1 Predicted transmembrane topologies of Orco with variable sites highlighted in red. The double line indicates the membrane region with extracellular and cytoplasmic sides labeled.

Figure S2 Ratio of the relative amino acid differences per domain averaged for Orco.

Table S1 Primers used in identification of Orco genes from mirid bug *A. suturalis, A. fasciaticollis*, and *L. pratensis*.

Table S2 Primers used in amplification of AlucOrco gene introns.

Table S3 Primers used in amplification of AlinOrco, AfasOrco and AsutOrco gene introns.

Table S4 Primers used in amplification of LpraOrco gene introns.

Table S5 Primers used in qPCR measurement.

Table S6 Identities of amino acid sequences among Orco genes from five mirid bugs.

Table S7 Orcos used in phyologenetic construction and sequence analysis.
