# Supplementary figures and images for: Characterization and Comparative Analysis of Olfactory Receptor Co-Receptor Orco Orthologs Among Five Mirid Bug Species
Source: Front Physiol. 2018 Mar 5;9:158. doi: 10.3389/fphys.2018.00158 (PMC5845112; doi:10.3389/fphys.2018.00158)

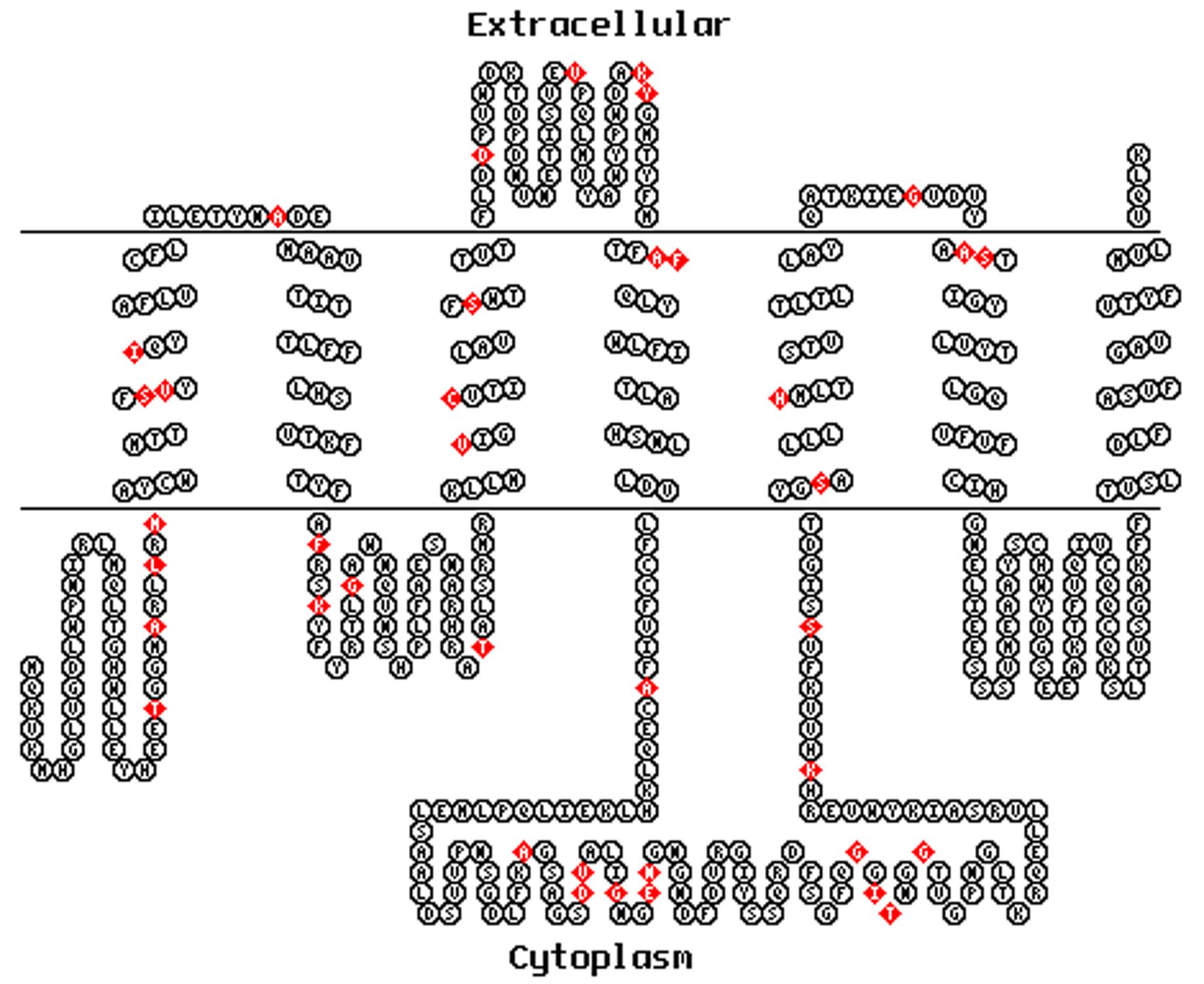

Supplement: Figure S1 — Predicted transmembrane topologies of Orco with variable sites highlighted in red. The double line indicates the membrane region with extracellular and cytoplasmic sides labeled. [file Image1.TIF]

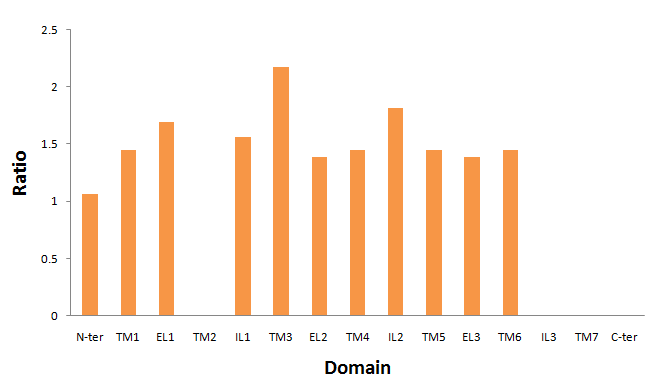

Supplement: Figure S2 — Ratio of the relative amino acid differences per domain averaged for Orco. [file Image2.TIF]
