# Supplementary material for: Characterization and Comparative Analysis of Olfactory Receptor Co-Receptor Orco Orthologs Among Five Mirid Bug Species
Source: Front Physiol. 2018 Mar 5;9:158. doi: 10.3389/fphys.2018.00158 (PMC5845112; doi:10.3389/fphys.2018.00158)
Supplement: Table S1 — Primers used in identification of Orco genes from mirid bug A. suturalis, A. fasciaticollis, and L. pratensis. [file Table1.DOCX]

***Supplementary Material***

**Characterization and comparative analysis of olfactory receptor coreceptor Orco** **orthologs among five mirid bug species**

***Qi Wang^1^, Qian Wang******^1, 3^, Yan-Le Zhou^1,4^,Shuang Shan^1^,Huan-Huan Cui^1^,Yong Xiao^1^, Kun Dong^1^, Adel Khashaveh^1^, Liang Sun^1, 2,*^, Yong-Jun Zhang^1, **^***

*^1^**State Key Laboratory for Biology of Plant Diseases and Insect Pests, Institute of Plant Protection, Chinese Academy of Agricultural Sciences, Beijing, China*

*^2^**Key Laboratory of Tea Quality and Safety Control, Ministry of Agriculture, Tea Research Institute, Chinese Academy of Agricultural Sciences, Hangzhou, China*

*^3^College of Horticulture and Plant Protection, Yangzhou University, Yangzhou, China*

*^4^DanDong Entry-Exit Inspection and Quarantine Bureau, Dandong, China*

***Correspondence:***

*^**^ Yong-Jun Zhang, E-mail:* [*yjzhang@ippcaas.cn*](mailto:yjzhang@ippcaas.cn)

*^*^ Liang Sun, E-mail:* [*liangsun@tricaas.com*](mailto:liangsun@tricaas.com)

**Supplementary Table S1** **Primers used in identification of Orco genes from mirid bug *A. suturalis*,*A. fasciaticollis* and *L. pratensis*.**

| **Primer name** | **Sequence (5’-3’)** |
| --- | --- |
| Degenerate forward primer | GCNATHAARTAYTGGGT |
| Degenerate reverse primer | TTYTGRCAYTGYTGRCAYAC |
| AsutOrco 5’-Race PCR GSP  AsutOrco 3’-Race PCR GSP | CGGATACCTGGTGTACACCCTCGGA  GCAGAAGGGCTGTGCCGTATGTGTC |
| AfasOrco 5’-Race PCR GSP  AfasOrco 3’-Race PCR GSP  LpraOrco 5’-Race PCR GSP  LpraOrco 3’-Race PCR GSP  Sequence verification forward primer  Sequence verification reverse primer | TACGGCACAGCCCTTCTGCTTCACAT  TACGGCACAGCCCTTCTGCTTCACAT  TGCTGACACACGATTTGGACGAACG  ATTGGCGACACTTATGGTTCGGCAC  ATGCAGAAAGTGAAGATGCA  TTATTTGAGCTGCACCAACAC |

Degeneration of oligonuletide mixtures: H=A/C/T, R=A/G, Y=C/T, V=A/C/G, N=A/C/G/T

**Supplementary Table S2 Primers used in amplification of AlucOrco gene introns**

| **Primer** | **Forward primer (5′-3′)** | **Reverse primer (5′-3′)** |
| --- | --- | --- |
| Intron1 | CAATACGCCTTCCTAGTCTGCTTC | GGAAAGTGACGGTCGTCCAA |
| Intron2 | GCACTGTCAAGGATGAGGAAGC | AGAGTTGGGGACGACCGAGT |
| Intron3 | ACTCTGGCGACTTGTTCAAAGCTGG | CTATAGATCCCCCTCACGTCAAAGTCAT |
| Intron4 | CCAAGGGGGAGTGGTGAATG | CAACATGTGAAGTAGAAGAGC |
| Intron5 | GTACGGTAACATTGACACTGCTCGCTTATC | CAAGTAGCCAATCGTGGAAGCTGCA |
| Intron6 | ATCGAAGCAGTCGACGTATATGCA | AACGTCTTGGCTTCTTCAGACC |

**Supplementary Table S3 Primers used in amplification of AlinOrco, AfasOrco and AsutOrco gene introns**

| **Primer** | **Forward primer (5′-3′)** | **Reverse primer (5′-3′)** |
| --- | --- | --- |
| Intron 1 | CGGATAGCTTACTGCTGGATGACCA | AATGCTGCTGCTCTGTGCCTCG |
| Intron 2 | CATTCTTGGACGATCCGGTTTGG | GCACTGAGTTCCATCAAAGGCTGG |
| Intron 3 | ACTCTGGCGACTTATTCAAATCAGG | CTGTATATCCCACGAACATCAAAATCAT |
| Intron 4 | CCAAGGAGGAATGACCAACG | CAACATGTGAAGCAGAAGGGC |
| Intron 5 | GTACTGTAACATTGACTCTACTCGCTTATC | CAGGTATCCGATCGTAGTAGATGCA |
| Intron 6 | ATCGAAGGTGTTGACGTATATGCA | AACGTCTTCGCCTCTTCAGATC |

**Supplementary Table S4 Primers used in amplification of LpraOrco gene introns**

| **Primer** | **Forward primer (5′-3′)** | **Reverse primer (5′-3′)** |
| --- | --- | --- |
| Intron 1-3 | TACATCCAATACGCCTTCCTAGTTTG | GCAACTCCTGTCTTTTGGTGAGTCC |
| Intron 4-5 | ATGGGAATGACTTTGACGTGCGAGG | AGGTTACAACAGCACCGAATACAGAAG |
| Intron 6-1 | CAGCTAGCACGATTGGCTACTTGG | GGTGCTAAGTTCTTCACTGTTTCCCT |
| Intron 6-2 | TTTCCCCAGTCCCCTAAC | TTATTTGAGCTGCACCAACACC |
| Intron 6-3 | TAAATTCCCTCCGCTCCA |  |
| Intron 6-4 | ACTTCTGCGTCGAATGTGT |  |
| Intron 6-5 | ATCGAAGGGGTCGACGTATATG |  |

**Supplementary Table S5 Primers used in qPCR measurement.**

| **Primer name** | **Forward primer(5′-3′)** | **Reverse primer(5′-3′)** |
| --- | --- | --- |
| *Asutβ-actin* | GCTACGTCGCTTTGGACTTC | CCTGAACCTTTCGTTTCCAA |
| *Alinβ-actin* | AAGGACCTGTACGCCAACAC | CCGGATTCGTCGTATTCTTG |
| *Afasβ-actin* | CATCTACGAGGGATACGCTCTC | TGGAGTTGTAAGTGGTTTCGTG |
| *Alucβ-actin* | GGATGCAGAAGGAAATCACC | GTGGAGAGAGAGGCGAGGAT |
| *Lpraβ-actin* | GCTGGTAGTAGACAATGGA | GTACGAGTCCTTCTGTCC |
| *AsutOrco* | CCTCAGCTCATGGTCTACGC | TTGAGTGTGCCAGCGTAATG |
| *AlinOrco* | ACGAAGAAACAGGTGGGATG | ATGAGGAAGCAGACGAGGAA |
| *AfasOrco* | ACGAAGAAACAGGTGGGATG | GAGGAAGCAGACAAGGAACG |
| *AlucOrco* | TCACGGAAACGAACTGATTG | CGTCTTGGCTTCTTCAGACC |
| *LpraOrco* | ACGTCAACGAGACCATTTCC | ACAACTGGAATGCGAAGGTC |

**Supplementary Table S6 Identities of amino acid sequences among Orco genes from five mirid bugs.**

|  | **AfasOrco** | **AsutOrco** | **AlinOrco** | **LprOrco** |
| --- | --- | --- | --- | --- |
| **AsutOrco** | 98.94 % |  |  |  |
| **AlinOrco** | 99.79 % | 99.15 % |  |  |
| **LprOrco** | 93.66 % | 92.23 % | 93.66 % |  |
| **AlucOrco** | 92.99 % | 92.57 % | 92.99 % | 96.41 % |

**Table S7 Orcos used in phyologenetic construction and sequence analysis**

| **Sequence name** | **Scientific name** | **accession number** | **Order** |
| --- | --- | --- | --- |
| AlinOrco | *Adelphocoris lineolatus* | KU523687 | Hemiptera |
| AfasOrco | *Adelphocoris fasciaticollis* | MF153393 | Hemiptera |
| AsutOrco | *Adelphocoris suturalis* | MF153394 | Hemiptera |
| AlucOrco | *Apolygus lucorum* | KC881255.1 | Hemiptera |
| LpraOrco | *Lygus pratensis* | MF153395 | Hemiptera |
| ClecOrco | Cimex lectularius | NP_001303637.1 | Hemiptera |
| NlugOrco | Nilaparvata lugens | XP_022185660.1 | Hemiptera |
| MperOrco | Myzus persicae | XP_022162891.1 | Hemiptera |
| ApisOrco | Acyrthosiphon pisum | XP_008188009.1 | Hemiptera |
| HhalOrco | Halyomorpha halys | XP_014279419.1 | Hemiptera |
| BtabOrco | Bemisia tabaci | XP_018916513.1 | Hemiptera |
| PsolOrco | Phenacoccus solenopsis | ANW12106.1 | Hemiptera |
| SaveOrco | Sitobion avenae | ACT37280.1 | Hemiptera |
| LhesOrco | Lygus hesperus | AFX73447.1 | Hemiptera |
| LlinOrco | Lygus lineolaris | AFX73448.1 | Hemiptera |
| CcapOrco | Ceratitis capitata | NP_001266301.1 | Diptera |
| ScalOrco | Stomoxys calcitrans | NP_001298174.1 | Diptera |
| DsuzOrco | Drosophila suzukii | NP_001315530.1 | Diptera |
| DwilOrco | Drosophila willistoni | XP_002072443.2 | Diptera |
| AaegOrco | Aedes aegypti | NP_001345400.1 | Diptera |
| DserOrco | Drosophila serrata | XP_020799410.1 | Diptera |
| MdomOrco | Musca domestica | XP_019891197.1 | Diptera |
| BdorOrco | Bactrocera dorsalis | XP_011203778.1 | Diptera |
| BoleOrco | Bactrocera oleae | XP_014092453.1 | Diptera |
| ZcucOrco | Zeugodacus cucurbitae | XP_011183998.1 | Diptera |
| DobsOrco | Drosophila obscura | XP_022220736.1 | Diptera |
| AalbOrco | Aedes albopictus | XP_019527302.1 | Diptera |
| BlatOrco | Bactrocera latifrons | XP_018789760.1 | Diptera |
| DbusOrco | Drosophila busckii | XP_017848171.1 | Diptera |
| DariOrco | Drosophila arizonae | XP_017856493.1 | Diptera |
| RzepOrco | Rhagoletis zephyria | XP_017470946.1 | Diptera |
| DmirOrco | Drosophila miranda | XP_017145273.1 | Diptera |
| DmelOrco | Drosophila melanogaster | NP_524235.2 | Diptera |
| DrhoOrco | Drosophila rhopaloa | XP_016982165.1 | Diptera |
| DeugOrco | Drosophila eugracilis | XP_017082279.1 | Diptera |
| DtakOrco | Drosophila takahashii | XP_017009993.1 | Diptera |
| DbipOrco | Drosophila bipectinata | XP_017098675.1 | Diptera |
| DeleOrco | Drosophila elegans | XP_017114693.1 | Diptera |
| DkikOrco | Drosophila kikkawai | XP_017023291.1 | Diptera |
| DficOrco | Drosophila ficusphila | XP_017044295.1 | Diptera |
| DbiaOrco | Drosophila biarmipes | XP_016948956.1 | Diptera |
| CstyOrco | Calliphora stygia | AID61201.1 | Diptera |
| CmegOrco | Chrysomya megacephala | AEA30004.2 | Diptera |
| CrufOrco | Chrysomya rufifacies | AFH96943.1 | Diptera |
| DpseOrco | Drosophila pseudoobscura | XP_001359364.3 | Diptera |
| LserOrco | Lucilia sericata | AEA30005.2 | Diptera |
| MdesOrco | Mayetiola destructor | AOT85634.1 | Diptera |
| SpyrOrco | Scaeva pyrastri | AOE48068.1 | Diptera |
| AfunOrco | Anopheles funestus | AIO10777.1 | Diptera |
| DgriOrco | Drosophila grimshawi | XP_001994023.1 | Diptera |
| CppaOrco | Culex pipiens pallens | AMQ13062.1 | Diptera |
| AgamOrco | Anopheles gambiae | XP_312379.3 | Diptera |
| CmarOrco | Clunio marinus | CRK97298.1 | Diptera |
| AgraOrco | Aldrichina grahami | ADN88092.1 | Diptera |
| DsecOrco | Drosophila sechellia | XP_002038406.1 | Diptera |
| DmojOrco | Drosophila mojavensis | XP_001998414.1 | Diptera |
| DperOrco | Drosophila persimilis | XP_002017182.1 | Diptera |
| DyakOrco | Drosophila yakuba | XP_002096053.1 | Diptera |
| HirrOrco | Haematobia irritans | ACF21678.1 | Diptera |
| DereOrco | Drosophila erecta | XP_001978924.1 | Diptera |
| DsimOrco | Drosophila simulans | XP_016033307.1 | Diptera |
| DhydOrco | Drosophila hydei | XP_023169755.1 | Diptera |
| DvirOrco | Drosophila virilis | XP_002056756.2 | Diptera |
| DanaOrco | Drosophila ananassae | XP_001953343.1 | Diptera |
| CquiOrco | Culex quinquefasciatus | ABB29301.1 | Diptera |
| AtraOrco | Amyelois transitella | NP_001299600.1 | Lepidoptera |
| PxylOrco | Plutella xylostella | NM_001309102.1 | Lepidoptera |
| PrapOrco | Pieris rapae | XP_022131026.1 | Lepidoptera |
| HarmOrco | Helicoverpa armigera | XP_021195606.1 | Lepidoptera |
| PmacOrco | Papilio machaon | XP_014363049.1 | Lepidoptera |
| EoblOrco | Ectropis obliqua | AKW50880.1 | Lepidoptera |
| PxutOrco | Papilio xuthus | XP_013167416.1 | Lepidoptera |
| PpolOrco | Papilio polytes | XP_013142400.1 | Lepidoptera |
| SlitOrco | Spodoptera litura | XP_022831582.1 | Lepidoptera |
| ObruOrco | Operophtera brumata | AJF20962.1 | Lepidoptera |
| MsexOrco | Manduca sexta | CUQ99422.1 | Lepidoptera |
| CpunOrco | Conogethes punctiferalis | ARO76408.1 | Lepidoptera |
| GmelOrco | Galleria mellonella | ALM30348.1 | Lepidoptera |
| EsemOrco | Eriocrania semipurpurella | ATV96621.1 | Lepidoptera |
| CmedOrco | Cnaphalocrocis medinalis | ALT31679.1 | Lepidoptera |
| ScerOrco | Sitotroga cerealella | AII15784.1 | Lepidoptera |
| LdisOrco | Lymantria dispar dispar | AHA50096.1 | Lepidoptera |
| CnigOrco | Cydia nigricana | AST36420.1 | Lepidoptera |
| CfagOrco | Cydia fagiglandana | AST36341.1 | Lepidoptera |
| HnubOrco | Hedya nubiferana | AST36292.1 | Lepidoptera |
| CpomOrco | Cydia pomonella | AFC91712.1 | Lepidoptera |
| AlepOrco | Athetis lepigone | AOE48007.1 | Lepidoptera |
| PoctOrco | Planotortrix octo | AJF23826.1 | Lepidoptera |
| AsegOrco | Agrotis segetum | AGS41440.1 | Lepidoptera |
| PsauOrco | Peridroma saucia | AQY16483.1 | Lepidoptera |
| AconOrco | Argyresthia conjugella | AEA76288.1 | Lepidoptera |
| SinfOrco | Sesamia inferens | AGY14565.1 | Lepidoptera |
| EposOrco | Epiphyas postvittana | ACJ12928.2 | Lepidoptera |
| BmorOrco | Bombyx mori | NP_001037060.1 | Lepidoptera |
| AdisOrco | Athetis dissimilis | ALJ33155.1 | Lepidoptera |
| HmroOrco | Heliconius melpomene rosina | AQQ73487.1 | Lepidoptera |
| MsepOrco | Mythimna separata | BAG71415.1 | Lepidoptera |
| OlatOrco | Ostrinia latipennis | BAH57974.1 | Lepidoptera |
| DhouOrco | Dendrolimus houi | AII01046.1 | Lepidoptera |
| OscaOrco | Ostrinia scapulalis | BAH57973.1 | Lepidoptera |
| HviaOrco | Heliothis viriplaca | AFI25169.1 | Lepidoptera |
| OfurOrco | Ostrinia furnacalis | AGG91643.1 | Lepidoptera |
| EhipOrco | Eogystia hippophaecolus | AOG12930.1 | Lepidoptera |
| CoblOrco | Ctenopseustis obliquana | AIT72022.1 | Lepidoptera |
| HassOrco | Helicoverpa assulta | ABU45983.2 | Lepidoptera |
| CsupOrco | Chilo suppressalis | AFQ94048.1 | Lepidoptera |
| OnubOrco | Ostrinia nubilalis | ADB89179.1 | Lepidoptera |
| DindOrco | Diaphania indica | BAG71418.1 | Lepidoptera |
| HzeaOrco | Helicoverpa zea | AAX14773.1 | Lepidoptera |
| OzagOrco | Ostrinia zaguliaevi | BAJ23265.1 | Lepidoptera |
| OpalOrco | Ostrinia palustralis | BAJ23262.1 | Lepidoptera |
| OzeaOrco | Ostrinia zealis | BAJ23260.1 | Lepidoptera |
| OovaOrco | Ostrinia ovalipennis | BAJ23264.1 | Lepidoptera |
| SexiOrco | Spodoptera exigua | AAW52583.1 | Lepidoptera |
| LdasOrco | Lymantria dispar asiatica | AHA50097.1 | Lepidoptera |
| DpplOrco | Danaus plexippus plexippus | OWR42934.1 | Lepidoptera |
| MbraOrco | Mamestra brassicae | AAS49925.1 | Lepidoptera |
| CherOrco | Ctenopseustis herana | AIT69913.1 | Lepidoptera |
| PnotOrco | Planotortrix notophaea | AET06159.1 | Lepidoptera |
| CsinOrco | Conopomorpha sinensis | ARQ32245.1 | Lepidoptera |
| PexcOrco | Planotortrix excessana | AET06156.1 | Lepidoptera |
| AperOrco | Antheraea pernyi | CAD88205.1 | Lepidoptera |
| HvisOrco | Heliothis virescens | CAD31851.1 | Lepidoptera |
| AcerOrco | Apis cerana | NP_001315406.1 | Hymenoptera |
| CcinOrco | Cephus cinctus | NP_001310774.1 | Hymenoptera |
| CsmaOrco | Ceratosolen solmsi marchali | NP_001292395.1 | Hymenoptera |
| ArosOrco | Athalia rosae | XP_012253637.1 | Hymenoptera |
| PgraOrco | Pseudomyrmex gracilis | XP_020289285.1 | Hymenoptera |
| ObirOrco | Ooceraea biroi | XP_011346854.1 | Hymenoptera |
| CfloOrco | Camponotus floridanus | XP_011253641.1 | Hymenoptera |
| HsalOrco | Harpegnathos saltator | XP_011139767.1 | Hymenoptera |
| NlecOrco | Neodiprion lecontei | XP_015513389.1 | Hymenoptera |
| DnovOrco | Dufourea novaeangliae | XP_015432571.1 | Hymenoptera |
| PdomOrco | Polistes dominula | XP_015184459.1 | Hymenoptera |
| DallOrco | Diachasma alloeum | XP_015126208.1 | Hymenoptera |
| PcanOrco | Polistes canadensis | XP_014601851.1 | Hymenoptera |
| DquaOrco | Dinoponera quadriceps | XP_014483273.1 | Hymenoptera |
| MdemOrco | Microplitis demolitor | XP_014295003.1 | Hymenoptera |
| TpreOrco | Trichogramma pretiosum | XP_014224691.1 | Hymenoptera |
| CoflOrco | Copidosoma floridanum | XP_014219015.1 | Hymenoptera |
| MphaOrco | Monomorium pharaonis | XP_012526645.1 | Hymenoptera |
| AfloOrco | Apis florea | XP_003690663.2 | Hymenoptera |
| OabiOrco | Orussus abietinus | XP_012273699.1 | Hymenoptera |
| BimpOrco | Bombus impatiens | XP_003494153.1 | Hymenoptera |
| LhumOrco | Linepithema humile | XP_012224569.1 | Hymenoptera |
| AcepOrco | Atta cephalotes | XP_012061929.1 | Hymenoptera |
| VemeOrco | Vollenhovia emeryi | XP_011867244.1 | Hymenoptera |
| WaurOrco | Wasmannia auropunctata | XP_011700383.1 | Hymenoptera |
| PbarOrco | Pogonomyrmex barbatus | XP_011632710.1 | Hymenoptera |
| PariOrco | Fopius arisanus | XP_011296908.1 | Hymenoptera |
| SinvOrco | Solenopsis invicta | XP_011164243.1 | Hymenoptera |
| AechOrco | Acromyrmex echinatior | XP_011057328.1 | Hymenoptera |
| CcosOrco | Cyphomyrmex costatus | XP_018396655.1 | Hymenoptera |
| TsepOrco | Trachymyrmex septentrionalis | XP_018347365.1 | Hymenoptera |
| TcorOrco | Trachymyrmex cornetzi | XP_018359353.1 | Hymenoptera |
| TzetOrco | Trachymyrmex zeteki | XP_018307006.1 | Hymenoptera |
| AcolOrco | Atta colombica | XP_018044487.1 | Hymenoptera |
| CcalOrco | Ceratina calcarata | XP_017890983.1 | Hymenoptera |
| HlabOrco | Habropoda laboriosa | XP_017793199.1 | Hymenoptera |
| EmexOrco | Eufriesea mexicana | XP_017766283.1 | Hymenoptera |
| McinOrco | Macrocentrus cingulum | AGI62937.2 | Hymenoptera |
| BterOrco | Bombus terrestris | XP_003402775.1 | Hymenoptera |
| CchlOrco | Campoletis chlorideae | AKO69815.1 | Hymenoptera |
| CjapOrco | Camponotus japonicus | BAO48211.1 | Hymenoptera |
| FariOrco | Fopius arisanus | XP_011296908.1 | Hymenoptera |
| CcorOrco | Ceratosolen cornutus | ACU31808.1 | Hymenoptera |
| CcunOrco | Chouioia cunea | AIY24336.1 | Hymenoptera |
| TsarOrco | Trichomalopsis sarcophagae | OXU18399.1 | Hymenoptera |
| AmelOrco | Apis mellifera | AHJ37468.1 | Hymenoptera |
| PpilOrco | Philotrypesis pilosa | ABY51616.1 | Hymenoptera |
| MquaOrco | Melipona quadrifasciata | KOX76355.1 | Hymenoptera |
| MpulOrco | Meteorus pulchricornis | AQN78403.1 | Hymenoptera |
| AbakOrco | Apocrypta bakeri | ABY51615.1 | Hymenoptera |
| AdorOrco | Apis dorsata | XP_006610550.1 | Hymenoptera |
| MmedOrco | Microplitis mediator | ABM05966.1 | Hymenoptera |
| ZnevOrco | Zootermopsis nevadensis | XP_021933609.1 | Blattaria |
| RspeOrco | Reticulitermes speratus | BAU20240.1 | Blattaria |
| DponOrco | Dendroctonus ponderosae | XP_019768125.1 | Coleoptera |
| TcasOrco | Tribolium castaneum | XP_008194693.1 | Coleoptera |
| OtauOrco | Onthophagus taurus | XP_022907054.1 | Coleoptera |
| AglaOrco | Anoplophora glabripennis | XP_018568191.1 | Coleoptera |
| AplaOrco | Agrilus planipennis | XP_018323449.1 | Coleoptera |
| AcorOrco | Anomala corpulenta | AKC58535.1 | Coleoptera |
| AquaOrco | Ambrostoma quadriimpressum | AJF94638.2 | Coleoptera |
| CbowOrco | Colaphellus bowringi | ALR72547.1 | Coleoptera |
| TmolOrco | Tenebrio molitor | AJO62219.1 | Coleoptera |
| RvulOrco | Rhynchophorus vulneratus | AOO35284.1 | Coleoptera |
| RferOrco | Rhynchophorus ferrugineus | AOO35283.1 | Coleoptera |
| HparOrco | Holotrichia parallela | AEG88961.1 | Coleoptera |
| HoblOrco | Holotrichia oblita | AEE69033.1 | Coleoptera |
| RdomOrco | Rhyzopertha dominica | AIX97139.1 | Coleoptera |
| NvesOrco | Nicrophorus vespilloides | XP_017785109.1 | Coleoptera |
| LdecOrco | Leptinotarsa decemlineata | XP_023027672.1 | Coleoptera |
| HpluOrco | Holotrichia plumbea | ADM35103.1 | Coleoptera |
| AtumOrco | Aethina tumida | XP_019869252.1 | Coleoptera |
| MaltOrco | Monochamus alternatus | AIX97092.1 | Coleoptera |
| AchiOrco | Anoplophora chinensis | AUF73041.1 | Coleoptera |
| SgreOrco | Schistocerca gregaria | AEX28371.1 | Orthoptera |
| LmigOrco | Locusta migratoria | AEX28370.1 | Orthoptera |
| PhumOrco | Pediculus humanus | ALX17413.1 | Anoplura |
